# Supplementary material for: Light and Shadows: Insights from Large-Scale Visual Screens for Arabidopsis Leaf Morphology Mutants
Source: Int J Mol Sci. 2025 Aug 28;26(17):8332. doi: 10.3390/ijms26178332 (PMC12427647; doi:10.3390/ijms26178332)
Supplement: Supplementary file 1 [file ijms-26-08332-s001.zip › ijms-3825166-supplementary.pdf]

Light and Shadows: Insights from Large-Scale Visual Screens for Arabidopsis Leaf Morphology Mutants

Lucía Juan-Vicente†, Alejandro Ruiz-Bayón†, and José Luis Micol

Instituto de Bioingeniería, Universidad Miguel Hernández, Campus de Elche, 03202 Elche, Spain

†These authors contributed equally to this work.

Supplementary Figures and Tables

Supplementary Material included in this file:

- Figures S1-S6
- Tables S1-S3

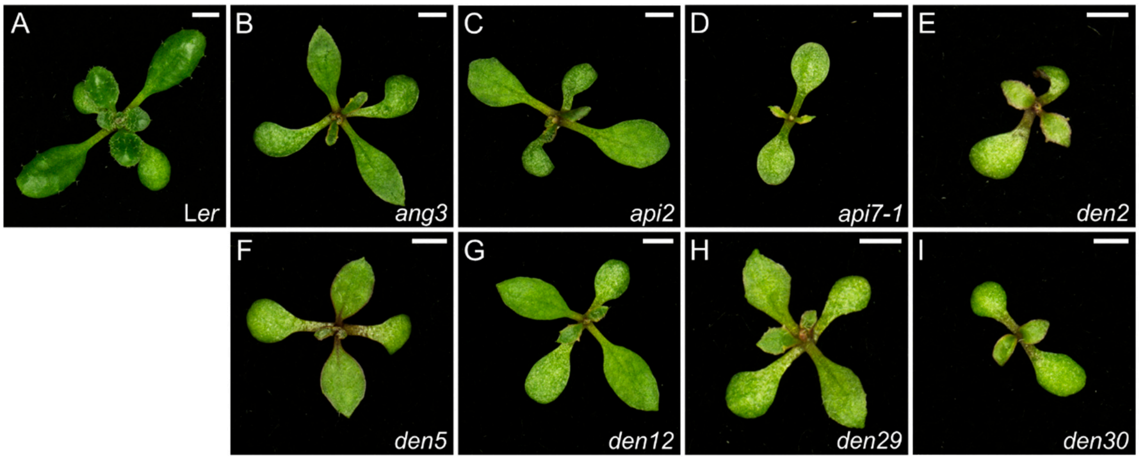

Figure S1. Leaf phenotypes of mutants with defects in translation. Rosettes of the wild-type L*er* (A), and the *ang3* (B), *api2* (C), *api7-1* (D), *den2* (E), *den5* (*rpl7b-1*) (F), *den12* (*rpl10ab-3*) (G), *den29* (*rps15ab-1 rpl28a-3*) (H), and *den30* (*rpl39c-1*) (I) homozygous mutants. Photographs were taken 15 das. Scale bars, 2 mm. The plants shown in this and the following supplementary figures share the same genotypes as those in the corresponding main figures but differ in age. We included this earlier time point to better illustrate the progression of the phenotype over time. These mutants were described in [54] (B), [55] (C), [58] (D), [57] (E), and [56] (F-I).

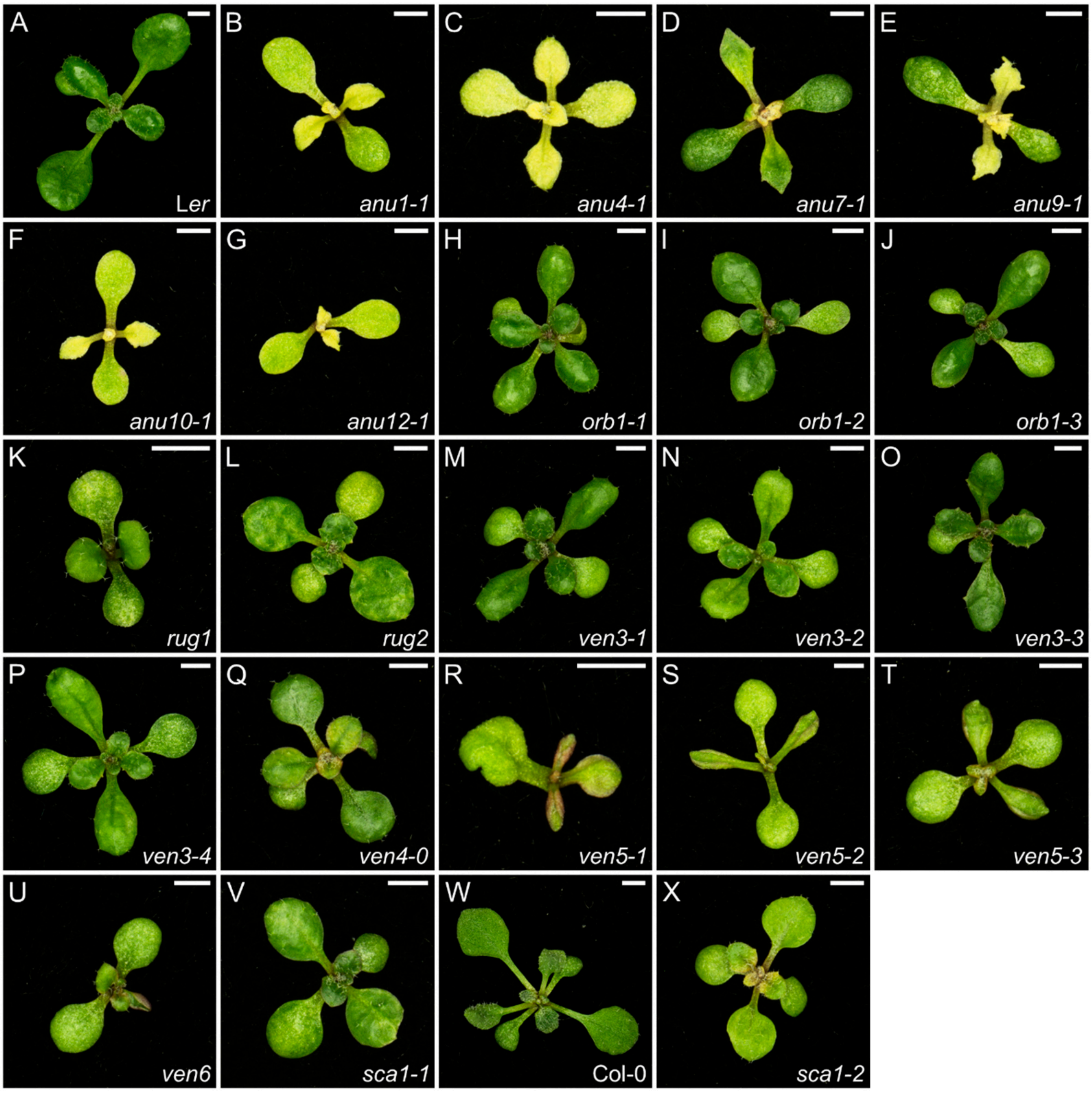

Figure S2. Leaf phenotypes of mutants with altered chloroplast biogenesis and function. Rosettes of the wild-types L*er* (A) and Col-0 (W), and the *anu1-1* (B), *anu4-1* (C), *anu7-1* (D), *anu9-1* (E), *anu10-1* (F), *anu12-1* (G), *orb1-1* (H), *orb1-2* (I), *orb1-3* (J), *rug1* (K), *rug2* (L), *ven3-1* (M), *ven3-2* (N), *ven3-3* (O), *ven3-4* (P), *ven4-0* (Q), *ven5-1* (*rer3-1*) (R), *ven5-2* (*rer3-2*) (S), *ven5-3* (*rer3-3*) (T), *ven6* (U), *sca1-1* (V), and *sca1-2* (X) homozygous mutants. Photographs were taken 15 das. Scale bars, 2 mm. These mutants were described in [65] (B, C), [72] (D), [65] (E), [71] (F), [65] (G), [90] (H-J), [78] (K), [81] (L), [84] (M-P), [91] (Q), [94] (R-T), [84] (U), [96] (V), and [96] (X).

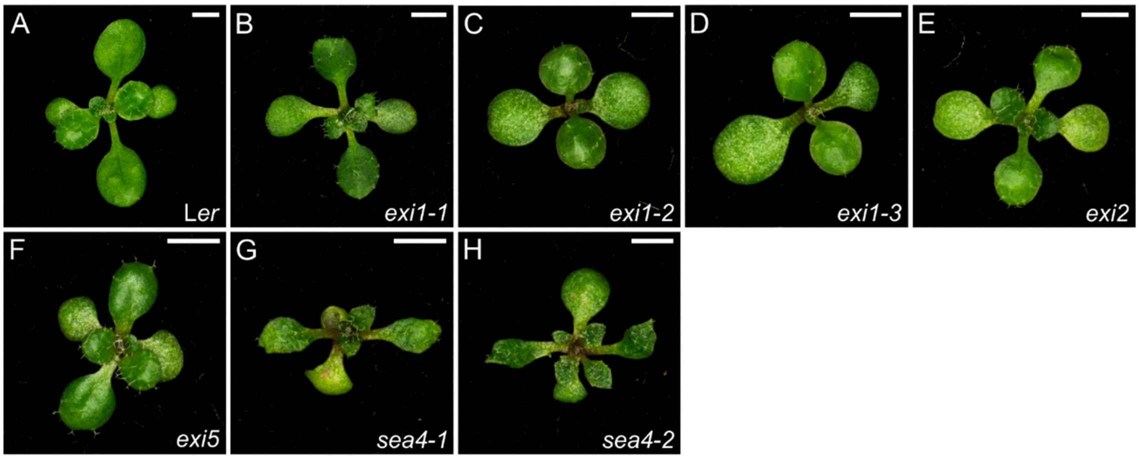

Figure S3. Leaf phenotypes of mutants with altered cell wall biosynthesis. Rosettes of the wild-type L*er* (A), and the *exi1-1* (B), *exi1-2* (C), *exi2* (D), *exi5* (E), *sea4-1* (F), and *sea4-2* (G) homozygous mutants. Photographs were taken 15 das. Scale bars, 2 mm. These mutants were described in [103] (B-F), and [100] (G, H).

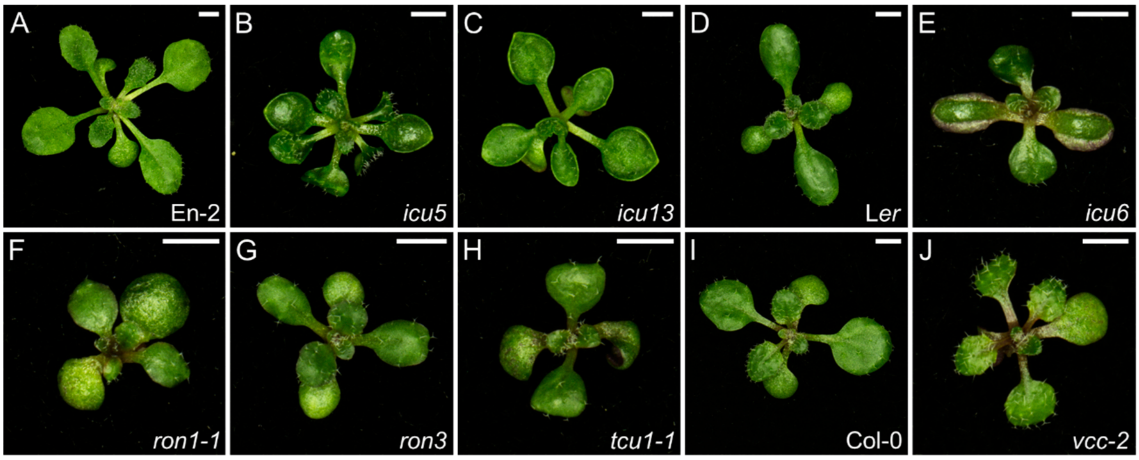

Figure S4. Leaf phenotypes of mutants with defects in auxin homeostasis. Rosettes of the wild-types En-2 (A), L*er* (D), and Col-0 (I), and the *icu5* (*shy2-10*) (B), *icu13* (C), *icu6* (E), *ron1-1* (F), *ron3* (G), *tcu1-1* (H), and *deal1-1* (*vcc-2*) (J) homozygous mutants. Photographs were taken 15 das. Scale bars, 2 mm. These mutants were described in [107] (B, C), [111] (E), [125] (F), [114] (G), [130] (H), and [138] (J).

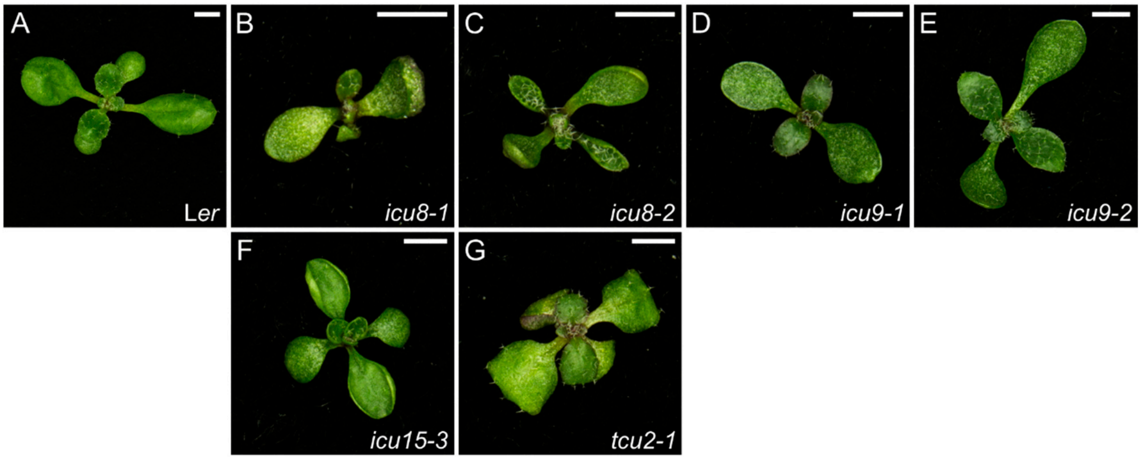

Figure S5. Leaf phenotypes of mutants altered in miRNA biogenesis and function. Rosettes of the wild-type L*er* (A), and the *icu8-1* (*hyl1-11*) (B), *icu8-2* (*hyl1-12*) (C), *icu9-1* (*ago1-51*) (D), *icu9-2* (*ago1-52*) (E), *icu15-3* (*hen1-13*) (F), and *tcu2-1* (G) homozygous mutants. Photographs were taken 15 das. Scale bars, 2 mm. These mutants were described in [143] (B-F), and [148] (G).

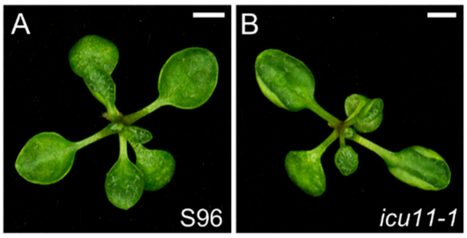

Figure S6. Leaf phenotypes of a mutant altered in its epigenetic machinery. Rosettes of the wild-type S96 (A) and the *icu11-1* homozygous mutant (B). Photographs were taken 15 das. Scale bars, 2 mm. This mutant was described in [163] (B).

Table S1. Mutants mentioned in this review

Process altered	Mutant name	Mutated gene				Some mutant phenotypic traits	Ref.
		Name	Other names	AGI code	Annotated function		
Translation	*ang3*	*ANG3*	*OLI7*[1], *RPL5B*[2], *UL18Y*[3]	At5g39740	Ribosomal protein RPL5B	Narrow leaves, reduced abaxial epidermal cell size, trichome density and primary root length	[54]
	api2	*API2*	*EL42Y*[4], *RPL36AB*[5]	At4g14320	Ribosomal protein RPL36aB	Pointed leaves, reduced plant height	[55]
	api7-1	*API7*	*ABCE2*[6], *RLI2*[7]	At4g19210	Cytoplasmic ribosome dissociation	Small rosette, short primary root, leaves pointed, toothed, pale, and with reduced photosynthetic pigments, venation pattern complexity and epidermal cell size	[58]
	den2	*DEN2*	*SMO4*[8]	At2g40430	Ribosome biogenesis factor involved in 5.8S rRNA maturation	Pointed and toothed leaves	[57]
	den5-2	*DEN5*	*RPL7B*[9], *UL30Y*[10]	At2g01250	Ribosomal protein RPL7B	Small, narrow, reticulate and pointed leaves, toothed first two leaves, presence of parallel veins on the proximal lamina, increased palisade mesophyll cell size and ploidy levels	[56]
	den12	*DEN12*	*PGY1*[11], *UL1Y*[12]	At2g27530	Ribosomal protein RPL10aB	Small, narrow, reticulate and pointed leaves, toothed first two leaves, increased palisade mesophyll cell size, increased ploidy levels	[56]
	den29	*DEN29*	*RPS15AB*[13], *US8MZ*[14], *EL28Z*[15]	At2g19720; At2g19730	Ribosomal proteins RPS15aB and RPL28A	Small, narrow, reticulate and pointed leaves, toothed first two leaves, parallel veins on the proximal lamina, disorganized palisade mesophyll, increased palisade mesophyll cell size and ploidy levels	[56]
	den30	*DEN30*	*EL39X*[16]	At4g31985	Ribosomal protein RPL39C	Small, narrow, reticulate and pointed leaves, toothed first two leaves, disorganized palisade mesophyll, increased palisade mesophyll cell size and ploidy levels	[56]

Table S1 (continued). Mutants mentioned in this review

Process altered	Mutant name	Mutated gene				Some mutant phenotypic traits	Ref.
		Name	Other names	AGI code	Annotated function		
Chloroplast biogenesis and function	*anu1-1*	*ANU1*	*SECA2*[17]	At1g21650	Movement of peptides through chloroplast membranes	Leaves with pale-yellow lamina and irregular leaf margins with prominent teeth	[65]
	anu4-1	*ANU4*	*PPI1*[18], *TOC33*[19]	At1g02280			
	anu7-1	*ANU7*	*EMB2737*[20], *EMB64*[21]	At5g53860	—	Pale-green leaves with prominent marginal teeth, reduced rosette size, plant height and chlorophyll and carotenoid levels, irregular and wide distribution of palisade mesophyll cell sizes	[72]
	anu9-1	*ANU9*	*ABCI11*[22], *NAP14*[23]	At5g14100	Metal homeostasis	Leaves with pale-yellow lamina and irregular leaf margins with prominent teeth	[65]
	anu10-1	*ANU10*	—	At1g28530	—	Pale-green rosettes, reduced plant height, rosette size and leaf lateral expansion, toothed leaf margin, increased size and reduced density of palisade mesophyll cells, accumulation of H_2O_2 in chloroplasts, reduced levels of chlorophyll and carotenoids, small and abnormally shaped chloroplasts, absent grana	[71]
	anu12-1	*ANU12*	*CLPR1*[24], *NCLPP5*[25], *SVR2*[26]	At1g49970	Protein degradation	Leaves with pale-yellow lamina and irregular leaf margins with prominent teeth	[65]
	orb1-1, *orb1-2*, and *orb1-3*	*ORB1*	*GLS1*[27], *FD-GOGAT1*[28]	At5g04140	Synthesis of glutamate and ammonium	Reduced rosette size, leaf lamina area, palisade mesophyll cell size and fresh and dry weight, pale green leaves with reduced chlorophyll and carotenoid levels	[90]
	rug1	*RUG1*	*HEMC*[30]	At5g08280	Tetrapyrrole biosynthesis pathway	Irregularly shaped leaves with hyponastic and protruding leaf lamina, reduced plant height, lesions in leaves, late flowering	[78]

Table S1 (continued). Mutants mentioned in this review

Process altered	Mutant name	Mutated gene				Some mutant phenotypic traits	Ref.
		Name	Other names	AGI code	Annotated function		
Chloroplast biogenesis and function	*rug2*	*RUG2*	*BSM*[29]	At4g02990	Homolog of the metazoan mitochondrial transcription termination factors (mTERFs)	Reduced organ size, leaves with both green and pale sectors (the latter containing sparsely packed mesophyll cells with abnormally shaped and fewer chloroplasts)	[81]
	sca1-1	*SCA1*	*RPS5*[31], *EMB3113*[32]	At2g33800	Ribosomal protein RPS5	Small rosette, pale-green leaves with reduced levels of chlorophylls *a* and *b*, reduced maximum efficiency of photosystem II, uneven leaf surface, prominent marginal teeth and reduced palisade mesophyll cell size	[96]
	ven3-1, ven3-2, ven3-3, and *ven3-4*	*VEN3*	*CARB*[33]	At1g29900	Carbamoyl phosphate synthetase (CPS) large subunit (CARB)	Reticulate, hyponastic first leaves, increased marginal tooth number in adult leaves, reduced size and number of palisade mesophyll cells	[84]
	ven4-0	*VEN4*	—	At5g40270	dNTPase (dNTP pool balance maintenance)	Pale and reticulate leaves with reduced chlorophyll content, chloroplasts reduced in size in palisade mesophyll with increased plastoglobuli number and poorly organized thylakoids	[91]
	ven5-1, ven5-2, and *ven5-3*	*VEN5*	*RER3*[34]	At3g08640	—	Reticulate, hyponastic and toothed leaves, disorganized mesophyll with increased air spaces, reduced leaf epidermal cell size and ploidy levels, embryonic patterning defects, perivascular accumulation of H_2O_2 and cell death	[94]
	ven6-1	*VEN6*	*CARA*[35]	At3g27740	CPS small subunit (CARA)	Reticulate, hyponastic first leaves, increased marginal tooth number in adult leaves, reduced size and number of palisade mesophyll cells	[84]

Table S1 (continued). Mutants mentioned in this review

Process altered	Mutant name	Mutated gene				Some mutant phenotypic traits	Ref.
		Name	Other names	AGI code	Annotated function		
Cell wall biosynthesis	*exi1-1* and *exi1-2*	*EXI1*	*CESA8*[36], *IRX1*[37], *LEW2*[38]	At4g18780	Subunit CESA8 of the cellulose synthase A complex	Leaves small, dark-green and with reduced cell sizes, reduced primary root and stem length, and flower and silique size and ploidy levels, collapsed xylem vessels in the stem	[103]
	exi2	*EXI2*	*CESA4*[39], *IRX5*[40], *NWS2*[41]	At5g44030	Subunit CESA4 of the cellulose synthase A complex	Small dark-green leaves, small primary root, stem, flowers and siliques, reduced leaf cell size and ploidy levels, collapsed stem xylem vessels	[103]
	exi5	*EXI5*	*CESA7*[42], *IRX3*[43], *MUR10*[44]	At5g17420	Subunit CESA7 of the cellulose synthase A complex		
	sea4-1 and *sea4-2*	*SEA4*	*KEU*[45], *SEC11*[46]	At1g12360	Sec1/Munc18 protein required for cytokinesis	Small rosettes, leaves serrated, small, wavy, and with a complex venation pattern, and exhibiting premature senescence	[100]
Auxin homeostasis	*deal1-1*	*DEAL1*	*VCC*[47]	At2g32280	Embryo provasculature development	Leaf margin with irregularly sized and unevenly spaced lobes and sinuses, leading to leaf bilateral symmetry breaking	[138]
	icu5	*ICU5*	*SHY2*[48], *IAA3*[49]	At1g04240	Negative regulator of auxin responses	Leaves hyponastic, with reduced leaf size, and increased number of free-ending veins	[107]
	icu6	*ICU6*	*AXR3*[50], *IAA17*[51]	At1g04250	Transcription regulator acting as a repressor of auxin-inducible gene expression	Hyponastic leaves, reduced adaxial pavement cell size, decreased palisade mesophyll cell size, enhanced responsiveness to auxin and increased number of palisade mesophyll cell layers	[111]

Table S1 (continued). Mutants mentioned in this review

Process altered	Mutant name	Mutated gene				Some mutant phenotypic traits	Ref.
		Name	Other names	AGI code	Annotated function		
Auxin homeostasis	*icu13*	*ICU13*	*AXR6*[52], *CUL1*[53], *ETA1*	At4g02570	Component of the SCF complex	Hyponastic leaves with reduced venation complexity, auxin responsiveness and adaxial pavement cell size, increased palisade and spongy mesophyll cell density and rosette leaf number at bolting, early flowering, reduced plant height and apical dominance	[107]
	ron1-1	*RON1*	*ALX8*[54], *FRY1*[55], *HOS2*[56], *OLD101*[57], *SAL1*, *SUPO1*[58]	At5g63980	Enzyme with inositol polyphosphate 1-phosphatase and 3′(2′),5′-bisphosphate nucleotidase activities	Rounded leaves, open venation pattern, reduced number of lateral roots, late flowering, loss of apical dominance	[125]
	ron3-1	*RON3*	*SIC*[59], *WARP2*[60]	At4g24500	Proline-rich protein involved in miRNA biogenesis, mRNA splicing and recycling of PIN1 and PIN2 auxin transporters	Reduced rosette leaf lamina area, late flowering, short roots with reduced hypergravitropic growth, reduced apical dominance	[114]
	tcu1-1	*TCU1*	*NUP58*[61]	At4g37130	Ortholog of nucleoporin of the Nup62 complex	Increased hypocotyl and petiole length, leaf lamina small, reticulate, and folded asymmetrically toward the abaxial surface with respect to the primary vein, early flowering	[130]
miRNA biogenesis and function	*icu8-1* and *icu8-2*	*ICU8*	*DRB1*[62], *HYL1*[63]	At1g09700	miRNA biogenesis and cleavage	Leaves hyponastic with poorly defined boundary between lamina and petiole and trichomes on the abaxial epidermis, compact inflorescences, poor fertility, late flowering, prematurely open flowers, incompletely fused carpels, small inflorescences, floral organs and fruits, reduced number of leaf stomata, simpler venation pattern	[143]

Table S1 (continued). Mutants mentioned in this review

Process altered	Mutant name	Mutated gene				Some mutant phenotypic traits	Ref.
		Name	Other names	AGI code	Annotated function		
miRNA biogenesis and function	*icu9-1* and *icu9-2*	*ICU9*	*AGO1*[64]	At1g48410	Component of the RISC	Most traits shared with the *icu8* mutants, except venation pattern complexity, which is increased compared to wild type	[143]
	icu15-1, icu15-2, and *icu15-3*	*ICU15*	*CRM2*[65], *HEN1*[66]	At4g20910	Component of the microRNA pathway	Most traits shared with the *icu8* mutants	[143]
Epigenetic machinery	*icu11-1*	*ICU11*	—	At1g22950	2-oxoglutarate/Fe(II)-dependent dioxygenase (histone modification)	Epinastic cotyledons, hyponastic leaves with reduced leaf palisade mesophyll cell size, early flowering, presence of unfertilized ovules in siliques	[163]

The full alternative names of the genes that appear in this table are: [1]*OLIGOCELLULA7*, [2]*RIBOSOMAL PROTEIN L5 B*, [3]*RIBOSOMAL PROTEIN UL18Y*, [4]*RIBOSOMAL PROTEIN EL42Y*, [5]*RIBOSOMAL PROTEIN L36A B*, [6]*ATP-BINDING CASSETTE E2*, [7]*RNASE L INHIBITOR PROTEIN 2*, [8]*SMALL ORGAN 4*, [9]*RIBOSOMAL PROTEIN L7 B*, [10]*RIBOSOMAL PROTEIN UL30Y*, [11]*PIGGYBACK1*, [12]*RIBOSOMAL PROTEIN UL1Y*, [13]*RIBOSOMAL PROTEIN S15A B*, [14]*RIBOSOMAL PROTEIN US8MZ*, [15]*RIBOSOMAL PROTEIN EL28Z*, [16]*RIBOSOMAL PROTEIN EL39X*, [17]*PROTEIN TRANSLOCASE SUBUNIT SECA2*, [18]*PLASTID PROTEIN IMPORT 1*, [19]*TRANSLOCON AT THE OUTER ENVELOPE MEMBRANE OF CHLOROPLASTS 33*, [20]*EMBRYO DEFECTIVE 2737*, [21]*EMBRYO DEFECTIVE 64*, [22]*ABC TRANSPORTER I FAMILY MEMBER 11*, [23]*NON-INTRINSIC ABC PROTEIN 14*, [24]*CLP PROTEASE PROTEOLYTIC SUBUNIT 1*, [25]*NUCLEAR CLPP 5*, [26]*SUPPRESSOR OF VARIEGATION 2*, [27]*GLUTAMATE SYNTHASE 1*, [28]*FERREDOXIN-DEPENDENT GLUTAMATE SYNTHASE 1*, [29]*BELAYA SMERT*, [30]*HYDROXYMETHYLBILANE SYNTHASE*, [31]*RIBOSOMAL PROTEIN* S5, [32]*EMBRYO DEFECTIVE 3113*, [33]*CARBAMOYL PHOSPHATE SYNTHETASE B*, [34]*RETICULATA-RELATED 3*, [35]*CARBAMOYL PHOSPHATE SYNTHETASE A*, [36]*CELLULOSE SYNTHASE 8*, [37]*IRREGULAR XYLEM 1*, [38]*LEAF WILTING 2*, [39]*CELLULOSE SYNTHASE 4*, [40]*IRREGULAR XYLEM 5*, [41]*NO WILT SYMPTOMS2*, [42]*CELLULOSE SYNTHASE 7*, [43]*IRREGULAR* XYLEM *3*, [44]*MURUS 10*, [45]*KEULE*, [46]Sec1/Munc18 protein gene *SEC11*, [47]*VASCULATURE COMPLEXITY AND CONNECTIVITY*, [48]*SHORT HYPOCOTYL 2*, [49]*INDOLE-3-ACETIC INDUCIBLE 3*, [50]*AUXIN RESISTANT 3*, [51]*INDOLE-3-ACETIC INDUCIBLE 17*, [52]*AUXIN RESISTANT 6*, [53]*CULLIN 1*, [54]*ALTERED EXPRESSION OF APX2 8*, [55]*FIERY1*, [56]*HIGH EXPRESSION OF OSMOTICALLY RESPONSIVE GENES 2*, [57]*ONSET OF LEAF DEATH 101*, [58]*SUPPRESSOR OF PIN1 OVEREXPRESSION 1*, [59]*SICKLE*, [60]*WARP ACUTE RESPONSE OF PRR7 2*, [61]*NUCLEOPORIN 58*, [62]*DSRNA-BINDING PROTEIN 1*, [63]*HYPONASTIC LEAVES 1*, [64]*ARGONAUTE 1*, [65]*CORYMBOSA 2*, [66]*HUA ENHANCER 1*, and [67]*NATB AUXILIARY SUBUNIT*.

Table S2. Mutants isolated in our screens or initially selected from the AIS collection

Phenotypic class	Mutant phenotype	Gene name	AGI gene code	Alternative gene names	Mutant allele	Origin
Angusta (Ang)	Narrow lamina	*ANG1*	ND		*ang1-1*	EMS
					ang1-2	EMS
		ANG2	ND		*ang2*	EMS
		ANG3	At5g39740	*OLI7*, *RPL5B*, *UL18Y*	*ang3*	EMS
		ANG4	At2g44950	*HUB1*, *RDO4*	*ang4*	EMS
		ANG5	ND		*ang5*	N241[a]
		ANG6	ND		*ang6*	N333[a]
Angulata (Anu)	Yellowish leaves with toothed margins	*ANU1*	At1g21650	*SECA2*	*anu1-1*	EMS
					anu1-2	EMS
		ANU2	At5g15450	*APG6*, *CLPB3*	*anu2*[b]	EMS
		ANU3	ND		*anu3*	EMS
		ANU4	At1g02280	*ATTOC33*, *PPI1*	*anu4*	EMS
		ANU5	ND		*anu5*	EMS
		ANU6	ND		*anu6*	EMS
		ANU7	At5g53860	*EMB2737*, *EMB64*	*anu7*	EMS
		ANU8	ND		*anu8*	EMS
		ANU9	At5g14100	*ABCI11*, *NAP14*	*anu9*	EMS
		ANU10	At1g28530		*anu10*	EMS
		ANU11	ND		*anu11*	EMS
		ANU12	At1g49970	*CLPR1*, *NCLPP5*, *SVR2*	*anu12*	EMS
Apiculata (Api)	Pointed lamina, with slightly incised margins	*API1*	ND		*api1*	EMS
		API2	At4g14320	*EL42Y*, *RPL36A*, *RPL36AB*	*api2*	EMS
		API3	ND		*api3*	EMS
		API4	ND		*api4*	EMS

Table S2 (continued). Mutants isolated in our screens or initially selected from the AIS collection

Phenotypic class	Mutant phenotype	Gene name	AGI gene code	Alternative gene names	Mutant allele	Origin
Apiculata (Api)	Pointed lamina, with slightly incised margins	*API5*	ND		*api5*	EMS
		API6	ND		*api6*	EMS
		API7	At4g19210	*ABCE2*, *RLI2*	*api7-1*	EMS
Asymmetric leaves (As)	Rounded lamina, with some degree of bilateral asymmetry and slightly revolute margins	*AS1*	At2g37630	*ATPHAN*, *LL2*, *MYB91*	*as1-11*[b]	EMS
					as1-12[b]	EMS
					as1-13[b]	EMS
					as1-14[b]	N321[a]
					as1-15[b]	N444[a]
					as1-16[b]	CS3240[a]
					as1-17[b]	CS3250[a]
					as1-18[b]	FN
		AS2	At1g65620		*as2-11*[b]	EMS
					as2-12[b]	N463[a]
					as2-13[b]	N230[a]
		AS3	ND		*as3-1*	FN
Calyciforme (Cay)	Dense rosette with some degree of fused leaves and cotyledons that sometimes result in a cup-shaped structure, stem fasciation, lower fertility and more pistils	*CAY*	ND		*cay*	N324[a]
Compact rosette (Cro)	Compact rosette with bushy inflorescences	*CRO1*	At2g38050	*DET2*, *DWF6*	*cro1-1*[b]	N303[a]
					cro1-2[b]	N319[a]
					cro1-3[b]	N352[a]

Table S2 (continued). Mutants isolated in our screens or initially selected from the AIS collection

Phenotypic class	Mutant phenotype	Gene name	AGI gene code	Alternative gene names	Mutant allele	Origin
Compact rosette (Cro)	Compact rosette with bushy inflorescences	*CRO1*	At2g38050	*DET2*, *DWF6*	*cro1-4*[b]	N359[a]
					cro1-5[b]	N387[a]
					cro1-6[b]	N389[a]
					cro1-7[b]	N390[a]
					cro1-8[b]	N416[a]
		CRO2	ND		*cro2-1*	N318[a]
					cro2-2	N355[a]
					cro2-3	N389[a]
		CRO3	At3g50660	*DWF4*, *CLM*, *CLAM*, *PSC1*, *SAV1*, *SNP2*	*cro3-1*[b,c]	N365[a]
					cro3-2[b,c]	N374[a]
					cro3-3[b]	N409[a]
		CRO4	ND		*cro4-1*	N317[a]
					cro4-2	N388[a]
					cro4-3	N417[a]
		CRO5	ND		*cro5*	N399[a]
	Compact rosette with nonbushy inflorescences	*CRO6*	ND		*cro6-1*	N398[a]
					cro6-2	N402[a]
		CRO7	ND		*cro7*	N331[a]
		CRO8	ND		*cro8-1*	N445[a]
		CRO9	ND		*cro9*	N403[a]
		CRO10	ND		*cro10*	N254[a]
		CRO11	ND		*cro11*	N334[a]
		CRO12	ND		*cro12*	N368[a]
		CRO13	ND		*cro13*	N462[a]

Table S2 (continued). Mutants isolated in our screens or initially selected from the AIS collection

Phenotypic class	Mutant phenotype	Gene name	AGI gene code	Alternative gene names	Mutant allele	Origin
Dentata (Dea)	Serrated margins	*DEA1*	ND		*dea1*	EMS
Denticulata (Den)	Pointed lamina, with toothed margins	*DEN1*	ND		*den1*	EMS
		DEN2	At2g40430	*SMO4*	*den2*	EMS
		DEN3	ND		*den3*	EMS
		DEN4	ND		*den4*	EMS
		DEN5	At2g01250	*UL30Y*, *RPL7B*	*den5-1*	EMS
					den5-2 (*rpl7b-1*)	EMS
		DEN6	ND		*den6-1*[d]	EMS
					den6-2	EMS
		DEN7	ND		*den7*	EMS
		DEN8	ND		*den8*	EMS
		DEN9	ND		*den9*	EMS
		DEN10	ND		*den10*	EMS
		DEN11	ND		*den11*	EMS
		DEN12	At2g27530	*PGY1*, *UL1Y*	*den12* (*rpl10ab-3*)	EMS
		DEN13	ND		*den13*	EMS
		DEN14	ND		*den14*	EMS
		DEN15	ND		*den15*	EMS
		DEN16	ND		*den16*	EMS
		DEN17	ND		*den17*	EMS
		DEN18	ND		*den18*	N243[a]
		DEN19	ND		*den19*	N316[a]
		DEN20	ND		*den20*	N320[a]

Table S2 (continued). Mutants isolated in our screens or initially selected from the AIS collection

Phenotypic class	Mutant phenotype	Gene name	AGI gene code	Alternative gene names	Mutant allele	Origin
Denticulata (Den)	Pointed lamina, with toothed margins	*DEN21*	ND		*den21*	N323[a]
		DEN22	ND		*den22*	N343[a]
		DEN23	ND		*den23*	N405[a]
		DEN24	ND		*den24*	N407[a]
		DEN25	ND		*den25*	N429[a]
		DEN26	ND		*den26*	N443[a]
		DEN27	ND		*den27-1*	N451[a]
					den27-2	N452[a]
		DEN28	ND		*den28*	CS3138[a]
		DEN29	At2g19720; At2g19730	*RPS15AB*, *US8MZ*; *EL28Z*	*den29* (*rps15ab-1 rpl28a-3*)	FN
		DEN30	Ar4g31985	*EL39X*	*den30* (*rpl39c-1*)	FN
		FASCIATA1 (*FAS1*)	At1g65470	*FUGU2*, *NFB2*	*fas1-11*[b]	N371[a]
		SERRATE (*SE*)	At2g27100		*se*[b,e]	CS3257[a]
		YELLOW INFLORESCENCE (*YI*)	ND		*yi-2*[b]	N447[a]
Elongata (Elo)	Narrow and elongated lamina and long petiole	*ELO1*	At3g11220	*ELP4*	*elo1*	EMS
		ELO2	At5g13680	*ABO1*, *ELP1*	*elo2*	EMS
		ELO3	At5g50320	*ELP3*, *EAST1*, *HAC8*, *HAG3*	*elo3*	EMS
		ELO4	At1g13870	*DRL1*	*elo4*	EMS

Table S2 (continued). Mutants isolated in our screens or initially selected from the AIS collection

Phenotypic class	Mutant phenotype	Gene name	AGI gene code	Alternative gene names	Mutant allele	Origin
Erosa (Ero)	Rounded lamina, with toothed margins	*ERO1*	ND		*ero1*	EMS
		ERO2	ND		*ero2*	EMS
		ERO3	ND		*ero3*	EMS
Exigua (Exi)	Small and dark leaves	*EXI1*	At4g18780	*CESA8*, *IRX1*, *LEW2*	*exi1-1*	EMS
					exi1-2	EMS
					exi1-3	EMS
		EXI2	At5g44030	*CESA4*, *IRX5*, *NWS2*	*exi2*	EMS
		EXI3	ND		*exi3*	EMS
		EXI4	ND		*exi4*	EMS
		EXI5	At5g17420	*CESA7*, *IRX3*, *MUR10*	*exi5*	EMS
		EXI6	ND		*exi6*	EMS
		EXI7	ND		*exi7*	EMS
		EXI8	ND		*exi8-1*	EMS
					exi8-2	EMS
					exi8-3	N438[a]
		EXI9	ND		*exi9-1*	FN
Filiforme (Flr)	Needle-shaped leaves	*FLR*	ND		*flr*	N325[a]
		FLAVODENTATA (*FLV*)	At4g18750	*DOT4*	*flv*[b,f]	CS3254[a]
Hemivenata (Hve)	Simple leaf venation pattern	*HVE1*	At2g02560	*CAND1*, *ETA2*, *TIP120*	*hve-1*	NV
Incurvata (Icu)	Margins curled upward	*ICU1*	At2g23380	*CLF*, *SDG1*, *SET1*	*icu1-1*	N313[a]
					icu1-2	N328[a]

Table S2 (continued). Mutants isolated in our screens or initially selected from the AIS collection

Phenotypic class	Mutant phenotype	Gene name	AGI gene code	Alternative gene names	Mutant allele	Origin
Incurvata (Icu)	Margins curled upward	*ICU1*	At2g23380	*CLF*, *SDG1*, *SET1*	*icu1-3*	N345[a]
					icu1-4	N346[a]
					icu1-5	N347[a]
					icu1-6	N350[a]
					icu1-7	N351[a]
					icu1-8	N419[a]
					icu1-9	EMS
					icu1-10	EMS
		ICU2	At5g67100		*icu2*	N329[a]
		ICU3	At3g05040	*HST1*	*icu3-1*	N314[a]
					icu3-2	EMS
		ICU4	At1g52150	*HB15*, *CNA*	*icu4-1*	N400[a]
					icu4-2	N401[a]
		ICU5	At1g04240	*IAA3*, *SHY2*	*icu5* (*shy2-10*)	N379[a]
		ICU6	At1g04250	*IAA17*, *AXR3*	*icu6*	EMS
		ICU7	ND		*icu7-1*	EMS
					icu7-2	EMS
		ICU8	At1g09700	*HYL1*	*icu8-1* (*hyl1-11*)	EMS
					icu8-2 (*hyl1-12*)	FN
		ICU9	At1g48410	*AGO1*	*icu9-1* (*ago1-51*)	EMS
					icu9-2 (*ago1-52*)	EMS
		ICU11	At1g22950		*icu11-1*	N242[a]
		ICU12	ND		*icu12*	N311[a]
		ICU13	At4g02570	*CUL1*, *AXR6*, *ETA1*	*icu13*	N349[a]

Table S2 (continued). Mutants isolated in our screens or initially selected from the AIS collection

Phenotypic class	Mutant phenotype	Gene name	AGI gene code	Alternative gene names	Mutant allele	Origin
Incurvata (Icu)	Margins curled upward	*ICU14*	ND		*icu14-1*	N330[a]
					icu14-2	N353[a]
		ICU15	At4g20910	*CRM2*, *HEN1*	*icu15-1* (*hen1-11*)	N357[a]
					icu15-2 (*hen1-12*)	N431[a]
					icu15-3 (*hen1-13*)	FN
Ondulata (Ond)	Undulated lamina	*OND1*	ND		*ond1*	EMS
		OND2	At1g69390	*ARC12*, *MINE1*	*ond2*[b]	EMS
		OND3	At5g42480	*ARC6*	*ond3*[b]	EMS
		OND4	ND		*ond4*[c]	EMS
Orbiculata (Orb)	Small, rounded, and yellowish leaves	*ORB1*	At5g04140	*GLS1*, *GLU1*, *GLUS*	*orb1-1*	EMS
					orb1-2	EMS
					orb1-3	EMS
		ORB2	ND		*orb2*	EMS
Rotunda (Ron)	Broad and rounded lamina	*RON1*	At5g63980	*ALX8*, *FRY1*, *HOS2*, *OLD101*, *SAL1*, *SUPO1*	*ron1-1*	EMS
		RON2	At4g32551	*LUG*	*ron2-1*	EMS
					ron2-2	EMS
					ron2-3	EMS
		RON3	At4g24500	*SIC*, *WARP2*	*ron3-1*	EMS
Rugosa (Rug)	Wrinkled lamina	*RUG1*	At5g08280	*HEMC*	*rug1*	EMS
		RUG2	At4g02990	*BSM*	*rug2*	EMS
Scabra (Sca)	Rounded and protruded lamina	*SCA1*	At2g33800	*EMB3113*, *PRPS5*, *RPS5*, *US5C*	*sca1*	EMS
		SCA2	ND		*sca2*	EMS
		SCA3	At2g24120	*PDE319*	*sca3*	EMS

Table S2 (continued). Mutants isolated in our screens or initially selected from the AIS collection

Phenotypic class	Mutant phenotype	Gene name	AGI gene code	Alternative gene names	Mutant allele	Origin
Scabra (Sca)	Rounded and protruded lamina	*SCA4*	ND		*sca4-1*	EMS
					sca4-2	EMS
					sca4-3	EMS
		SCA5	At5g20040	*IPT9*	*sca5*[b]	EMS
Serrata (Sea)	Small leaves with strongly serrated margins	*SEA1*	ND		*sea1*	EMS
		SEA2	At2g43710	*FAB2*, *LDW1*, *SSI2*	*sea2*[b]	EMS
		SEA3	ND		*sea3*	EMS
		SEA4	At1g12360	*KEU*, *SEC11*	*sea4-1*	EMS
					sea4-2	EMS
Transcurvata (Tcu)	Margin obliquely revolute	*TCU1*	At4g37130	*NUP58*	*tcu1*	EMS
		TCU2	At1g03150	*NAA20*	*tcu2*	EMS
		TCU3	ND		*tcu3*	EMS
		TCU4	ND		*tcu4*	N423[a]
Tortifolia (Tor)	Late flowering, petioles and leaves turn progressively counterclockwise	*TOR1*	At4g27060	*CN*, *SPR2*	*tor1-1*[b,g]	N378[a]
Ultracurvata (Ucu)	Lamina spirally rolled downward	*INVALIDA* (*INL*)	ND		*inl*[e]	CS3397[a]
		UCU1	At4g18710	*SK2*, *BIN2*, *DWF12*	*ucu1-1*	EMS
					ucu1-2	EMS
					ucu1-3	EMS
		UCU2	At3g21640	*FKBP42*, *TWD1*	*ucu2-1*	FN
Venosa (Ven)	Conspicuous venation; some lines displaying incised margins	*VEN1*	At5g05730	*AMT1*, *ASA1*, *JDL1*, *TRP5*, *WEI2*	*ven1*[b]	EMS
		VEN2	At2g37860	*RETICULATA* (*RE*)	*ven2-1*	EMS
					ven2-2	EMS

Table S2 (continued). Mutants isolated in our screens or initially selected from the AIS collection

Phenotypic class	Mutant phenotype	Gene name	AGI gene code	Alternative gene names	Mutant allele	Origin
Venosa (Ven)	Conspicuous venation; some lines displaying incised margins	*VEN3*	At1g29900	*CARB*	*ven3-1*	EMS
					ven3-2	EMS
					ven3-3	EMS
					ven3-4	EMS
		VEN4	At5g40270		*ven4-0*	EMS
		VEN5	At3g08640	*RER3*	*ven5-1* (*rer3-1*)	EMS
					ven5-2 (*rer3-2*)	EMS
					ven5-3 (*rer3-3*)	EMS
		VEN6	At3g27740	*CARA*	*ven6*	EMS

This table includes all the mutants that we isolated after ethyl methanesulfonate (EMS) and fast-neutron (FN) mutagenesis [13,24] as well as [a]those that we initially selected from the Arabidopsis Information Service (AIS) collection [25], some of which were ultimately not studied by us. [b]Mutants that were not studied further, or were analyzed only to determine their allelism with previously published mutants exhibiting similar leaf phenotypes; in some cases, the causal genes had been or were later identified by other authors. [c]Mutants isolated by [173]. [d]Candidate region described in [174]. [e-g] Mutants isolated by [e,f]Rédei ([e][95]; [f]cited in the ABRC catalog 1995), and [g][175]. Some information, such as gene names and AGI codes, is repeated from Supplementary Table S1; however, it is also included here to facilitate comprehension. ND, not determined, as the corresponding mutant was not subjected to genetic analysis and the causative gene responsible for its phenotype was therefore not identified. NV, natural variant.

Table S3. Double mutant combinations of mutations (1 and 2) that we obtained (1) with those obtained by other authors (2)

Gene	Mutation 1[a]	Mutation 2[a]	Annotated function of the protein encoded by the gene harboring mutation 2	Leaf phenotype	Ref.
ANU7	*anu7-1*	*gun1-1*	Chloroplast-localized pentatricopeptide-repeat protein involved in regulation of nuclear gene expression	Synergistic	[72]
	anu7-1	*gun2-1*	Plastid heme oxygenase necessary for phytochrome chromophore biosynthesis and coupling nuclear gene expression to the state of chloroplast	Additive	[72]
	anu7-1	*gun3-1*	Phytochromobilin synthase involved in tetrapyrrole phytochrome biosynthesis and coupling nuclear gene expression to the state of chloroplast	Additive	[72]
	anu7-1	*gun4-1*	Porphyrin-binding protein required for chlorophyll accumulation and coupling the expression of nuclear genes to the state of chloroplast	Additive	[72]
API2	*api2*	*as1-1*	MYB-domain protein involved in the specification of the leaf proximodistal axis	Additive	[55]
	api2	*as2-1*	AS2/LOB protein family involved in the epigenetic repression of abaxial identity genes	Synergistic	[55]
	api2	*rpl36aA*	Member of the Zinc-binding ribosomal protein family	Synergistic	[55]
API7	*api7-1*	*as1-1*	MYB-domain protein involved in specification of the leaf proximodistal axis	Additive	[58]
	api7-1	*as2-1*	AS2/LOB protein family involved in the epigenetic repression of abaxial identity genes	Synergistic	[58]
AS1	*as1-14*	*as2-12*	AS2/LOB protein family involved in the epigenetic repression of abaxial identity genes	Synergistic	[25]
	as1-14	*as2-13*	AS2/LOB protein family involved in the epigenetic repression of abaxial identity genes	Synergistic	[25]
DEN5	*den5*	*as1-14*	MYB-domain protein involved in the specification of the leaf proximodistal axis	Additive	[56]
	den5	*as2-1*	AS2/LOB protein family involved in the epigenetic repression of abaxial identity genes	Synergistic	[56]
DEN12	*den12*	*as1-14*	MYB-domain protein involved in the specification of the leaf proximodistal axis	Additive	[56]
	den12	*as2-1*	AS2/LOB protein family involved in the epigenetic repression of abaxial identity genes	Synergistic	[56]
DEN29	*den29*	*as1-14*	MYB-domain protein involved in the specification of the leaf proximodistal axis	Additive	[56]
	den29	*as2-1*	AS2/LOB protein family involved in the epigenetic repression of abaxial identity genes	Synergistic	[56]
DEN30	*den30*	*as1-14*	MYB-domain protein involved in the specification of the leaf proximodistal axis	Additive	[56]
	den30	*as2-1*	AS2/LOB protein family involved in the epigenetic repression of abaxial identity genes	Synergistic	[56]
ELO1	*elo1*	*elo3*	Subunit of the Elongator histone acetyl transferase complex	Synergistic	[158]
ELO2	*elo2*	*elo1*	Subunit of the Elongator histone acetyl transferase complex	Synergistic	[158]
	elo2	*drl1-2*	Homolog of yeast TOT4/KTI12 that physically interacts with the Elongator complex	Synergistic	[158]

Table S3 (continued). Double mutant combinations of mutations (1 and 2) that we obtained (1) with those obtained by other authors (2)

Gene	Mutation 1[a]	Mutation 2[a]	Annotated function of the protein encoded by the gene harboring mutation 2	Leaf phenotype	Ref.
ELO3	*elo3*	*elo2*	Subunit of the Elongator histone acetyl transferase complex	Synergistic	[158]
ELO4	*elo4*	*elo1*	Subunit of the Elongator histone acetyl transferase complex	Synergistic	[158]
EXI1	*exi1-2*	*aba1-1*	Zeaxanthin epoxidase catalyzing the first step of the ABA biosynthesis pathway	Additive	[103]
	exi1-2	*aba2-13*	Short-chain dehydrogenase catalyzing the conversion of zanthoxin to ABA-aldehyde	Additive	[103]
EXI2	*exi2*	*aba1-1*	Zeaxanthin epoxidase catalyzing the first step of the ABA biosynthesis pathway	Additive	[103]
	exi2	*aba2-13*	Short-chain dehydrogenase catalyzing the conversion of zanthoxin to ABA-aldehyde	Additive	[103]
EXI5	*exi5*	*aba1-1*	Zeaxanthin epoxidase catalyzing the first step of the ABA biosynthesis pathway	Additive	[103]
	exi5	*aba2-13*	Short-chain dehydrogenase catalyzing the conversion of zanthoxin to ABA-aldehyde	Additive	[103]
ICU1	*clf-18*	*icu2-1*	Putative catalytic subunit of DNA polymerase α that interacts with TFL2 and CLF	Synergistic	[157]
	clf-18	*hst-5*	Importin/exportin family member involved in miRNA transport and timing of shoot maturation	Additive	[157]
	clf-18	*icu4-1*	Class III HD-ZIP protein critical for vascular development	Additive	[157]
	clf-18	*icu5*	SHY2/IAA3; regulates multiple auxin responses in roots	Additive	[157]
ICU2	*icu2-1*	*hst-5*	Importin/exportin family member involved in miRNA transport and timing of shoot maturation	Additive	[157]
	icu2-1	*icu4-1*	Class III HD-ZIP protein critical for vascular development	Additive	[157]
	icu2-1	*icu5*	SHY2/IAA3; regulates multiple auxin responses in roots	Additive	[157]
	icu2-1	*ft-1*	Together with LFY, promotes flowering and is antagonistic with TFL1	Synergistic	[161]
	icu2-1	*ag-1*	Floral homeotic gene encoding a MADS domain transcription factor that specifies floral meristem and carpel and stamen identity	Synergistic	[161]
	icu2-1	*ap1-1*	Floral homeotic gene encoding a MADS domain protein homologous to SRF transcription factors that specifies floral meristem and sepal identity	Additive	[161]
	icu2-1	*ap3-4*	Floral homeotic gene encoding a MADS domain protein homologous to SRF transcription factors that specifies petal and stamen identities	Additive	[161]
	icu2-1	*pi-1*	Floral homeotic gene encoding a MADS domain transcription factor that is required for the specification of petal and stamen identities	Additive	[161]
	icu2-1	*clf-2*	Polycomb-group gene encoding a PRC2 core component	Synergistic	[161]

Table S3 (continued). Double mutant combinations of mutations (1 and 2) that we obtained (1) with those obtained by other authors (2)

Gene	Mutation 1[a]	Mutation 2[a]	Annotated function of the protein encoded by the gene harboring mutation 2	Leaf phenotype	Ref.
ICU2	*icu2-1*	*tfl2-2*	Protein localized preferentially to euchromatic regions involved in euchromatin organization	Synergistic	[161]
	icu2-1	*emf2-5*	Polycomb-group gene encoding a PRC2 core component	Synergistic	[161]
	icu2-1	*fas1-1*	Subunit of the heterotrimeric complex chromatin assembly factor-1 (CAF-1)	Synergistic	[161]
	icu2-1	*fas2-1*	CAF-1 subunit	Synergistic	[161]
ICU3	*hst-5*	*icu4-1*	Class III HD-ZIP protein critical for vascular development	Synergistic	[157]
	hst-5	*icu5*	SHY2/IAA3; regulates multiple auxin responses in roots	Additive	[157]
ICU4	*icu4-1*	*icu5*	SHY2/IAA3; regulates multiple auxin responses in roots	Additive	[157]
	icu4-1	*hst-1*	Importin/exportin family member involved in miRNA transport and timing of shoot maturation	Synergistic	[176]
	icu4-1	*kan1-2*	KANADI transcription factor required for abaxial identity in leaves and carpels	Additive	[176]
	icu4-1	*pkl-1*	SWI/SWF nuclear-localized chromatin remodeling facor	Additive	[176]
	icu4-1	*crc-1*	Putative transcription factor specifying abaxial cell fate	Synergistic	[176]
ICU5	*icu5*	*eta1*	CULLIN1; is a component of SCF ubiquitin ligase complexes involved in mediating responses to auxin and jasmonic acid	Additive	[107]
ICU6	*icu6*	*eta1*	CULLIN1; is a component of SCF ubiquitin ligase complexes involved in mediating responses to auxin and jasmonic acid	Synergistic	[107]
	icu6	*axr1-12*	Subunit of the RUB1 activating enzyme that regulates the protein degradation activity of SCF proteins	Synergistic	[111]
	icu6	*as1-11*	See the double mutants obtained with *api2*	Synergistic	[111]
	icu6	*as2-11*	AS2/LOB protein family involved in the epigenetic repression of abaxial identity genes	Synergistic	[111]

Table S3 (continued). Double mutant combinations of mutations (1 and 2) that we obtained (1) with those obtained by other authors (2)

Gene	Mutation 1[a]	Mutation 2[a]	Annotated function of the protein encoded by the gene harboring mutation 2	Leaf phenotype	Ref.
ICU11	*icu11-1*	*cp2-1*	Closest paralog of ICU11, with which it functions redundantly in regulating histone modifications	Synergistic	[163]
	icu11-1	*cp2-2*	Closest paralog of ICU11, with which it functions redundantly in regulating histone modifications	Synergistic	[163]
	icu11-1	*cp2-3*	Closest paralog of ICU11, with which it functions redundantly in regulating histone modifications	Synergistic	[163]
	icu11-1	*gis-5*	Catalytic subunit of DNA polymerase δ that deposits epigenetic marks	Synergistic	[163]
	icu11-1	*icu2-1*	Putative catalytic subunit of DNA polymerase α that interacts with TFL2 and CLF	Synergistic	[163]
	icu11-1	*clf-2*	PcG gene encoding a PRC2 core component	Synergistic	[163]
	icu11-1	*tfl2-2*	Protein localized preferentially to euchromatic regions involved in euchromatin organization	Synergistic	[163]
	icu11-1	*ebs-1*	Chromatin remodeling factor that regulates flowering time	Synergistic	[163]
	icu11-1	*fas1-1*	Subunit of the heterotrimeric complex CAF-1	Synergistic	[163]
	icu11-1	*dml2-3*	DNA glycosylase involved in maintaining methylation marks	Additive	[165]
	icu11-1	*dml3-1*	DNA glycosylase involved in maintaining methylation marks	Additive	[165]
	icu11-1	*dnmt2-2*	DNA methyltransferase homolog	Additive	[165]
	icu11-1	*drm1-2*	Methyltransferase involved in *de novo* DNA methylation and maintenance of asymmetric methylation	Additive	[165]
	icu11-1	*drm2-2*	Putative DNA methyltransferase	Additive	[165]
	icu11-1	*mbd10-1*	Protein containing a methyl-CpG-binding domain	Additive	[165]
	icu11-1	*met1-4*	Cytosine methyltransferase	Additive	[165]
	icu11-1	*ros1-4*	DNA demethylase that demethylates gene promoters and represses its silencing	Additive	[165]
	icu11-1	*ros3-2*	RNA-binding protein involved in DNA demethylation	Additive	[165]
	icu11-1	*vim3-2*	E3 ubiquitin ligase with a PHD domain and two RING domains	Additive	[165]
	icu11-1	*atx1-2*	Histone-lysine N-methyltransferase involved in trimethylating H3K4	Additive	[165]
	icu11-1	*atxr6*	H3K27 monomethylation that is required for chromatin structure and gene silencing	Additive	[165]
	icu11-1	*atxr7-1*	H3K4 methylation and transcriptional activation of *FLC*	Additive	[165]
	icu11-1	*hac1-3*	Co-activator of transcription with histone acetyl-transferase activity	Additive	[165]
	icu11-1	*hda6-6*	RPD3-like histone deacetylase	Additive	[165]
	icu11-1	*hda6-7*	RPD3-like histone deacetylase	Additive	[165]
	icu11-1	*ham1-1*	Histone acetyltransferase that primarily acetylates histone H4	Additive	[165]

Table S3 (continued). Double mutant combinations of mutations (1 and 2) that we obtained (1) with those obtained by other authors (2)

Gene	Mutation 1[a]	Mutation 2[a]	Annotated function of the protein encoded by the gene harboring mutation 2	Leaf phenotype	Ref.
ICU13	*icu13*	*rce1-10*	RUB1 conjugating enzyme that conjugates CUL1 and is involved in auxin response	Synergistic	[107]
	icu13	*axr1-12*	RUB1 activating enzyme subunit that regulates DCF protein degradation activity	Synergistic	[107]
	icu13	*csn5a-2*	COP9 complex subunit involved in protein deneddylation and required for recovery of AUX/IAA repressor levels	Synergistic	[107]
	icu13	*hve-2*	Homolog of CAND1 required for SCF function and appears to modulate SCF complex cycling	Synergistic	[107]
	icu13	*icu5*	SHY2/IAA3; regulates multiple auxin responses	Additive	[107]
	icu13	*axr2-1*	IAA7 represses auxin-inducible gene expression and is degraded in the presence of auxin	Additive	[107]
	icu13	*axr3-3*	IAA17 represses auxin-inducible gene expression and is degraded in the presence of auxin	Additive	[107]
	icu13	*icu6*	IAA17 represses auxin-inducible gene expression and is degraded in the presence of auxin	Additive	[107]
	icu13	*bdl*	IAA12 plays a role in auxim-mediated processes of embry apical-basal patterning	Synergistic	[107]
	icu13	*mp-S319*	Transcription factor mediating embryo axis formation and vascular development	Synergistic	[107]
	icu13	*iaa12-1*	IAA12 plays a role in auxim-mediated processes of embry apical-basal patterning	Suppression	[107]
RON1	*ron1-2*	*cvp1-3*	Sterol methyltransferase SMT2 involved in sterol biosynthesis	Synergistic	[125]
	ron1-2	*cvp2-1*	Inositol polyphosphate 5'-phosphatase required for recruitment of cells into developing vascular tissue	Synergistic	[125]
	ron1-2	*hve-2*	Homolog of CAND1 required for SCF function and appears to modulate SCF complex cycling	Synergistic	[125]
	ron1-2	*axr1-2*	Subunit of the RUB1 activating enzyme that regulates the protein degradation activity of SCF proteins	Synergistic	[125]
RON3	*ron3-1*	*ron1-1*	Inositol polyphosphate 1-phosphatase and 3'(2'),5'-biphosphate nucleotidase involved in degradation of small RNAs that regulates hypocotyl elongation	Synergistic	[114]
	ron3-2	*rcn1*	PP2A regulatory subunit A	Synergistic	[114]
RUG2	*rug2*	*sca3-1*	Nucleus-encoded chloroplast RNA polymerase RpoTp	Synergistic	[81]
SCA1	*sca1-1*	*as1-1*	MYB-domain protein involved in specification of the leaf proximodistal axis	Additive	[96]
	sca1-1	*as2-1*	AS2/LOB protein family involved in the epigenetic repression of abaxial identity genes	Synergistic	[96]
	sca1-2	*as2-1*	AS2/LOB protein family involved in the epigenetic repression of abaxial identity genes	Synergistic	[96]
SCA3	*sca3-2*	*rpoT;2*	NEP targeted to mitochondria and chloroplasts	Synergistic	[177]

Table S3 (continued). Double mutant combinations of mutations (1 and 2) that we obtained (1) with those obtained by other authors (2)

Gene	Mutation 1[a]	Mutation 2[a]	Annotated function of the protein encoded by the gene harboring mutation 2	Leaf phenotype	Ref.
SEA4	*sea4-1*	*sec1b-1*	Sec1/Munc18 (SM) protein member of KEULE gene family	Synergistic	[100]
	sea4-1	*sec1b-2*	SM protein member of the KEULE gene family	Synergistic	[100]
	sea4-1	*sec6-2*	Exocyst protein involved in tethering vesicles to the plasma membrane	Synergistic	[100]
	sea4-1	*sec6-3*	Exocyst protein involved in tethering vesicles to the plasma membrane	Synergistic	[100]
	sea4-1	*syp21*	Syntaxin member of the SYP2 gene family	Synergistic	[100]
	sea4-1	*syp132^T*	Syntaxin member of SYP13 gene family involved in the endocytosis of plasma membrane H^+-ATPase proteins	Synergistic	[100]
	sea4-1	*kn^{X37-2}*	Syntaxin member of SYP11 protein localized in the trans-Golgi network	Synergistic	[100]
	sea4-2	*sec1b-1*	SM protein member of KEULE gene family	Synergistic	[100]
	sea4-2	*sec1b-2*	SM protein member of KEULE gene family	Synergistic	[100]
	sea4-2	*sec6-2*	Exocyst protein involved in tethering vesicles to the plasma membrane	Synergistic	[100]
	sea4-2	*sec6-3*	Exocyst protein involved in tethering vesicles to the plasma membrane	Synergistic	[100]
	sea4-2	*syp21*	Syntaxin member of the SYP2 gene family	Synergistic	[100]
	sea4-2	*syp132^T*	Syntaxin member of SYP13 gene family involved in the endocytosis of plasma membrane H^+-ATPase proteins	Synergistic	[100]
	sea4-2	*kn^{X37-2}*	Syntaxin member of SYP11 protein localized in the trans-Golgi network	Synergistic	[100]
TCU1	*tcu1-2*	*nup54-1*	Nucleoporin	Synergistic	[130]
	tcu1-2	*nup54-2*	Nucleoporin	Synergistic	[130]
	tcu1-2	*nup62-1*	Nucleoporin that appears to be a major negative regulator of auxin signalling	Synergistic	[130]
	tcu1-1	*nup62-2*	Nucleoporin that appears to be a major negative regulator of auxin signalling	Synergistic	[130]
	tcu1-2	*nup62-2*	Nucleoporin that appears to be a major negative regulator of auxin signalling	Synergistic	[130]
	tcu1-1	*sar1-4*	Nucleoporin that regulates CONSTANS protein stability	Synergistic	[130]
	tcu1-2	*sar1-4*	Nucleoporin that regulates CONSTANS protein stability	Synergistic	[130]
	tcu1-1	*sar3-3*	Nucleoporin required for the activation of downstream defense pathways	Synergistic	[130]

Table S3 (continued). Double mutant combinations of mutations (1 and 2) that we obtained (1) with those obtained by other authors (2)

Gene	Mutation 1[a]	Mutation 2[a]	Annotated function of the protein encoded by the gene harboring mutation 2	Leaf phenotype	Ref.
TCU1	*tcu1-1*	*hst-21*	Importin/exportin family member involved in miRNA transport and timing of shoot maturation	Synergistic	[130]
	tcu1-1	*imn*	Homolog of human importin β1	Synergistic	[130]
	tcu1-1	*icu5*	SHY2/IAA3 regulates multiple auxin responses	Synergistic	[130]
	tcu1-1	*axr1-12*	Subunit of the RUB1 activating enzyme that regulates the protein degradation activity of SCF proteins	Synergistic	[130]
	tcu1-1	*axr3-3*	IAA17; represses auxin-inducible gene expression and is degraded in the presence of auxin	Synergistic	[130]
TCU2	*tcu2-1*	*zll-2*	AGO10; involved in relative organization of central zone and peripheral zone cells in meristems	Synergistic	[148]
UCU1	*ucu1-1*	*axr2-1*	IAA7; represses auxin-inducible gene expression and is degraded in the presence of auxin	Synergistic	[178]
	ucu1-1	*shy2-3*	SHY2/IAA3; regulates multiple auxin responses	Synergistic	[178]
	ucu1-1	*det2-1*	Similar to mammalian steroid-5-alpha-reductase involved in brassinolide biosynthetic pathway	Additive	[178]
	ucu1-1	*dim1-1*	Ca^{2+}-dependent calmodulin binding protein involved in the conversion of the early brassinosteroid precursor 24-methylenecholesterol to campesterol	Additive	[178]
	ucu1-1	*bri1-1*	Leucine-rich repeat receptor kinase involved in brassinosteroid signal transduction	Synergistic	[178]
UCU2	*ucu2-1*	*ucu1-1*	BIN2; member of the ATSK family that functions in the cross-talk between auxin and brassinosteroid signaling pathways	Additive[b]	[179]
	ucu2-1	*ucu1-2*	BIN2; member of the ATSK family that functions in the cross-talk between auxin and brassinosteroid signaling pathways	Additive[b]	[179]
	ucu2-1	*ucu1-3*	BIN2; member of the ATSK family that functions in the cross-talk between auxin and brassinosteroid signaling pathways	Synergistic	[179]
	ucu2-1	*dim1-1*	Ca^{2+}-dependent calmodulin binding protein involved in the conversion of the early brassinosteroid precursor 24-methylenecholesterol to campesterol	Additive	[179]
	ucu2-1	*det2-1*	Similar to mammalian steroid-5-alpha-reductase involved in brassinolide biosynthetic pathway	Additive	[179]

Table S3 (continued). Double mutant combinations of mutations (1 and 2) that we obtained (1) with those obtained by other authors (2)

Gene	Mutation 1[a]	Mutation 2[a]	Annotated function of the protein encoded by the gene harboring mutation 2	Leaf phenotype	Ref.
VCC	*vcc-2*	*pin1-1*	Auxin efflux carrier involved in shoot, root and leaf development	Additive	[138]
	vcc-2	*cuc2-3*	Transcriptional activator of the NAC gene family required for leaf serrations and the control of initiation of axillary meristems	Additive	[138]
	vcc-2	*CUC2g-m4*	Transcriptional activator of the NAC gene family required for leaf serrations and the control of initiation of axillary meristems	Additive	[138]
VEN2	*re-3*	*cue1-6*	Phosphoenolpyruvate (PEP)/phosphate antiporter located in the inner envelope of plastids.	Synergistic	[180]
	re-3	*dov1*	Glutamine 5-phosphoribosylpyrophosphate amidotransferase involved in differential development of vascular-associated cells	Additive	[180]
	re-3	*rer1-1*	Closest paralog of VEN2	Synergistic	[94]
	re-4	*rer1-1*	Closest paralog of VEN2	Synergistic	[94]
	re-3	*rer1-2*	Closest paralog of VEN2	Synergistic	[94]
	re-4	*rer1-2*	Closest paralog of VEN2	Synergistic	[94]
	re-3	*rer1-3*	Closest paralog of VEN2	Synergistic	[94]
	re-4	*rer1-3*	Closest paralog of VEN2	Synergistic	[94]
	re-4	*rer3-3*	Alphavirus core family protein (DUF3411); paralog of VEN2	Additive	[94]
	re-3	*ven3-1*	CARB; subunit of carbamoyl phosphate synthetase for arginine biosynthesis	Additive	[94]
	re-3	*ven6-1*	CARA; carbamoyl phosphate synthetase for arginine biosynthesis	Additive	[94]
	re-4	*noa1-2*	Protein with similarity to bacterial YqeH GTPase required for proper ribosome assembly and plastid biogenesis	Additive	[94]
	re-3	*as1-14*	MYB-domain protein involved in specification of the leaf proximodistal axis	Additive	[94]
	re-4	*as2-11*	AS2/LOB protein family involved in the epigenetic repression of abaxial identity genes	Additive	[94]
VEN3	*ven3-1*	*ven6-1*	CARA; Subunit of carbamoyl phosphate synthetase for arginine biosynthesis	Synergistic	[84]
	ven3-2	*ven6-1*	CARA; Subunit of carbamoyl phosphate synthetase for arginine biosynthesis	Synergistic	[84]
VEN4	*ven4-0*	*tso2-1*	dNTP biosynthesis	Synergistic	[91]
	ven4-0	*dov1*	Purine nucleotide biosynthesis in chloroplasts	Synergistic	[91]

Table S3 (continued). Double mutant combinations of mutations (1 and 2) that we obtained (1) with those obtained by other authors (2)

Gene	Mutation 1[a]	Mutation 2[a]	Annotated function of the protein encoded by the gene harboring mutation 2	Leaf phenotype	Ref.
VEN5	*rer3-1*	*ven6-1*	CARA; Subunit of carbamoyl phosphate synthetase for arginine biosynthesis	Additive	[94]
	rer3-2	*ven3-1*	CARB; Subunit of carbamoyl phosphate synthetase for arginine biosynthesis	Additive	[94]
	rer3-2	*as2-11*	AS2/LOB protein family involved in the epigenetic repression of abaxial identity genes	Additive	[94]
	rer3-2	*noa1-2*	Protein with similarity to bacterial YqeH GTPase required for proper ribosome assembly and plastid biogenesis	Additive	[94]
	rer3-3	*rer4-1*	Putative UvrABC system C protein (DUF3411); paralog of VEN5	Additive	[94]
	rer3-3	*rer1-1*	Integral component of chloroplast envelope membrane; paralog of VEN5	Additive	[94]
	rer3-3	*cue1-6*	Phosphoenolpyruvate (PEP)/phosphate antiporter located in the inner envelope of plastids.	Additive	[94]
	rer3-3	*as1-14*	MYB-domain protein involved in specification of the leaf proximodistal axis	Additive	[94]
	rer3-3	*rer4-1*	Putative UvrABC system C protein (DUF3411); paralog of VEN5	Additive	[94]
	rer3-3	*rer6-1*	Putative DUF399 family protein; paralog of VEN5	Additive	[94]
	rer3-3	*rer1-2*	Paralog of VEN5, integral component of chloroplast envelope membrane	Additive	[94]

Ref., reference. [a]Since we intercrossed many of the mutations that we isolated, some of them appear both in the columns headed as “Mutation 1” and “Mutation 2”. [b]Due to the strong phenotype of *ucu1-1* and *ucu1-2*, it was not possible to determine whether the phenotype of *ucu1 ucu2* double mutants was synergistic or merely additive.
